# CAD/CAM and Digital Flow Training in Dental Hygiene Vocational Education

**DOI:** 10.4317/jced.63411

**Published:** 2025-11-30

**Authors:** Iván Carrasco-Guardiola, Manuel Pabón-Carrasco, Ana Orozco-Varo, Juan José Segura-Egea, Jenifer Martín-González

**Affiliations:** 1 Department of Stomatology, Endodontic Section, School of Dentistry, University of Sevilla, Sevilla, Spain; 2 Department of Nursing, School of Nursing, University of Sevilla, Sevilla, Spain

## Abstract

**Background:**

The integration of digital technologies, such as CAD/CAM systems, into dental practice has significantly transformed both clinical workflows and educational methodologies. However, their incorporation into vocational training programs for Dental Hygiene remains limited. This study aimed to assess the level of familiarity, training, and perceptions related to CAD/CAM technologies and digital workflows among students enrolled in a Higher Technician in Dental Hygiene program.

**Material and Methods:**

A cross-sectional survey was conducted with 327 students enrolled in in-person, blended, and online modalities at a vocational training center in Seville, Spain. Data were collected using a validated 34-item questionnaire addressing demographics, digital literacy, and the perceived educational value of digital tools. Statistical analysis was performed using the Chi-square test and Cramér's V.

**Results:**

The sample was predominantly female (91.4%), with participants ranging in age from 18 to over 50 years. While 90.5% reported satisfaction with their academic program, 42.8% were unfamiliar with the term digital workflow, and 53.8% did not recognize the concept of CAD/CAM. Furthermore, 64.2% had received no prior training in digital workflows. Nonetheless, 89.3% of respondents expressed interest in incorporating such technologies into their curriculum, and 81.7% believed that 3D digital tools would be beneficial to their training. Statistically significant associations were observed between digital proficiency and positive attitudes toward the educational and clinical relevance of digital dentistry.

**Conclusions:**

These findings reveal a considerable gap in current curricula, but also a strong willingness among students to adopt technological advancements. Integrating digital workflows and CAD/CAM systems into vocational training programs appears essential to better prepare students and align educational offerings with modern professional standards in dentistry.

## Introduction

The advent of digital dentistry has brought about a paradigm shift in dental practice, particularly through the integration of computer-aided design and manufacturing (CAD/CAM) systems. These technologies are increasingly used in clinical settings, offering advantages such as improved treatment accuracy and reduced patient waiting times (1). As a result, errors associated with conventional methods have been minimized, contributing to better clinical outcomes and enhanced patient experiences (2). The incorporation of CAD/CAM technologies into dental education has been positively received by students, who report favorable perceptions regarding their usefulness and educational value. For instance, a recent study conducted in Egypt found that 73.6% of students demonstrated high levels of awareness and interest in digital dentistry, highlighting the need for more practical training in these systems (3). Similarly, other research has shown that students consider CAD/CAM a valuable tool for enhancing their clinical competencies (4). Despite this enthusiasm, faculty members-although possessing solid theoretical knowledge-report limited use of CAD/CAM technologies in their teaching practices (5). These findings underscore the need to strengthen both hands-on training for students and professional development for instructors to ensure effective integration of CAD/CAM systems into dental curricula. In the context of digital dentistry education, integrating these technologies into academic programs is essential to adequately prepare future dental professionals (6). Research suggests that current students, who have grown up immersed in digital environments, demonstrate a greater ease and willingness to adopt new technologies compared to previous generations, who often encounter more challenges in adapting to digital advancements (7). A recent survey of 60 faculty members across Spain revealed that while 71.7% reported familiarity with digital technologies, only 23.3% incorporated them into undergraduate programs, and just 35% into postgraduate courses. The intraoral scanner was identified as the most commonly used tool; however, many institutions still lack the necessary equipment. The steep learning curve and high associated costs were cited as major limiting factors, reinforcing the urgent need to improve digital workflow training at the undergraduate level (8). Regarding dental hygiene students, a study conducted in South Korea with 635 participants found that only 40% were familiar with CAD/CAM technology, and just 3.6% had hands-on experience using it. However, those who received practical training reported significantly more positive perceptions. Moreover, students in more advanced stages of their education expressed greater support for incorporating CAD/CAM into the curriculum (9). The integration of digital dentistry into vocational training programs is an emerging trend aimed at equipping future Higher Technicians specializing in Dental Hygiene and Dental Prosthetics with the technological competencies required to meet the evolving demands of the profession (10). However, current vocational curricula often fall short of aligning with best practices, with limited instruction in digital technologies. Recent studies conducted among undergraduate students in Colombia highlight the urgent need to incorporate advanced tools-such as CAD/CAM systems, intraoral scanners, and computer-aided design software-into dental education. These findings reinforce the necessity of aligning dental training with ongoing technological advancements in the field (11). Accordingly, the aim of this study is to evaluate the level of familiarity, training, and perceptions related to CAD/CAM technologies and digital workflows among students enrolled in higher vocational education programs in Dental Hygiene.

## Material and Methods

1. Study Design and Ethics A cross-sectional, questionnaire-based study was conducted at the Ilerna Vocational Training Centre, located in the Parque Alcosa district of Seville, Spain. The objective was to assess students' perceptions and knowledge regarding digital workflows and CAD/CAM technologies among those enrolled in a Higher Technician in Dental Hygiene program. Participants from all three learning modalities-on-site, blended, and online-were included. The survey was completed either in person or via mobile devices while present at the training center. Ethical procedures followed the principles outlined in the American Psychological Association (APA) Code of Ethics, particularly with regard to informed consent, confidentiality, and respect for participants. No personally identifiable data were collected. This study was approved by the Research Ethics Committee of the University of Seville under internal protocol code SICEIA 2025-1291. 2. Inclusion and Exclusion Criteria Eligibility criteria included students who were actively enrolled in a Higher Vocational Training program in Dental Hygiene at the time of the study, regardless of their educational modality-face-to-face, blended, or online. The inclusion of students from all modalities aimed to ensure broad representation across diverse learning contexts. Exclusion criteria encompassed individuals who were not enrolled in the Dental Hygiene program, as well as those who had already completed the corresponding academic cycle prior to survey administration. These criteria ensured that only current students with direct exposure to the program's curricular content were included in the analysis. 3. Questionnaire The data collection instrument was a validated questionnaire consisting of 34 closed-ended items, developed based on prior studies related to digital education in dentistry. The questionnaire was structured into several domains: sociodemographic information (gender, age, academic year), digital competence (self-perceived proficiency with digital tools), and academic interest (satisfaction with the chosen program). Additionally, the survey included items assessing knowledge and perceptions of digital technologies, including CAD, CAM, and integrated CAD/CAM systems, as well as familiarity with digital workflows and the perceived educational value of 3D technologies. Participants were also asked about their views on the effectiveness of digital tools-such as tablets and extraoral scanners-in dental education, along with their potential clinical benefits when used in conjunction with CAD/CAM systems. 4. Statistical Analysis A total of 326 valid responses were obtained out of 327 submitted questionnaires. The sample included both male and female students, with a higher proportion of female respondents. Participants were enrolled in either the first or second academic year of the Dental Hygiene program. Their ages ranged from 18 to over 50 years, providing a broad demographic representation. Socioeconomic context was also considered, as the training center is located in one of Seville's districts with the lowest per capita income, according to data from the Spanish Tax Agency. All data were analyzed using IBM SPSS Statistics version 29.0 (IBM Corp., Armonk, NY, USA). Descriptive statistics were used to report frequencies and percentages for each item. Inferential analyses were conducted using the Chi-square test to examine associations between categorical variables, and Cramér's V was calculated to assess the strength of those associations. Statistical significance was set at p &lt; 0.05.

## Results

A total of 326 students participated in the study, all of whom met the predefined inclusion and exclusion criteria. Most respondents were female (91.4%), while male participants comprised a smaller proportion of the sample. Participants' ages ranged from 18 to over 50 years, reflecting a broad age distribution within the study population. In terms of academic satisfaction, 90.5% (n = 296) reported that they were engaged in studies they found enjoyable, indicating a high level of satisfaction with their chosen academic path. Regarding academic progression, 58.4% (n = 190) of students were enrolled in the first year of the Dental Hygiene program, while 41.6% (n = 136) were in their second year. These figures provide a representative overview of the student population across different stages of the training cycle. Concerning familiarity with the term digital workflow, 42.8% (n = 140) of respondents indicated that they were unfamiliar with the concept, while 21.1% (n = 69) selected the option "Don't know / No answer" (NS/NC), (Table 1).

Table 1

A clear understanding of the concept of digital workflow is essential in the context of digital dentistry. When asked about prior training in digital workflows, 64.2% (n = 210) of students reported not having received any instruction in this area, while 15.9% (n = 52) selected the option "Don't know / No answer" (NS/NC). The detailed distribution of responses is presented in Table 2.

Table 2

**Figure 1:**
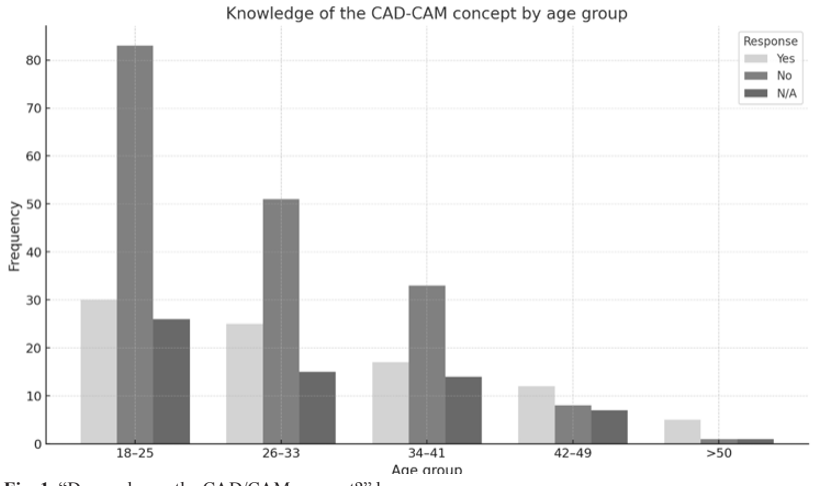
“Do you know the CAD/CAM concept?” by age range.

The study revealed that 53.8% (n = 176) of students reported being unfamiliar with the term CAD/CAM, while 19.0% (n = 62) selected the option "Don't know / No answer" (NS/NC). The gender distribution showed no statistically significant differences in CAD/CAM knowledge between male and female participants. However, age-based analysis indicated that the highest prevalence of limited knowledge was observed among students aged 18 to 41 years. A detailed breakdown of these results by age group is presented in Figure 1.

1

Regarding the integration of digital workflows into the academic training of Dental Hygiene students, 89.3% (n = 292) of participants expressed interest in incorporating this content into the curriculum. The detailed distribution of responses is shown in Table 3.

Table 3

The present study also examined students' perceptions regarding the usefulness of 3D technology in the academic training of those specializing in Dental Hygiene. The results showed that a majority of participants-59.9% (n = 196)-considered this technology to be beneficial for their education. Further analysis by age group and sex revealed no significant differences, suggesting a general acceptance of 3D technology across the study population. Detailed responses regarding the perceived benefits of tools such as digital tablets and CAD/CAM systems in enhancing classroom learning are presented in Table 4.

Table 4

**Figure 2:**
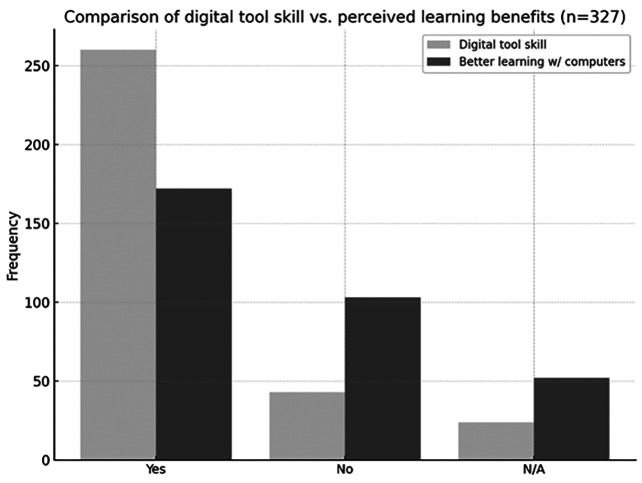
Comparison of digital tool proficiency versus perceived learning benefits.

Analysis of the relationship between the use of computer tools and learning in the classroom revealed that 44.3% (n = 145) of participants considered that the use of computers facilitates their learning, representing the majority view (Fig. 2).

2

**Figure 3:**
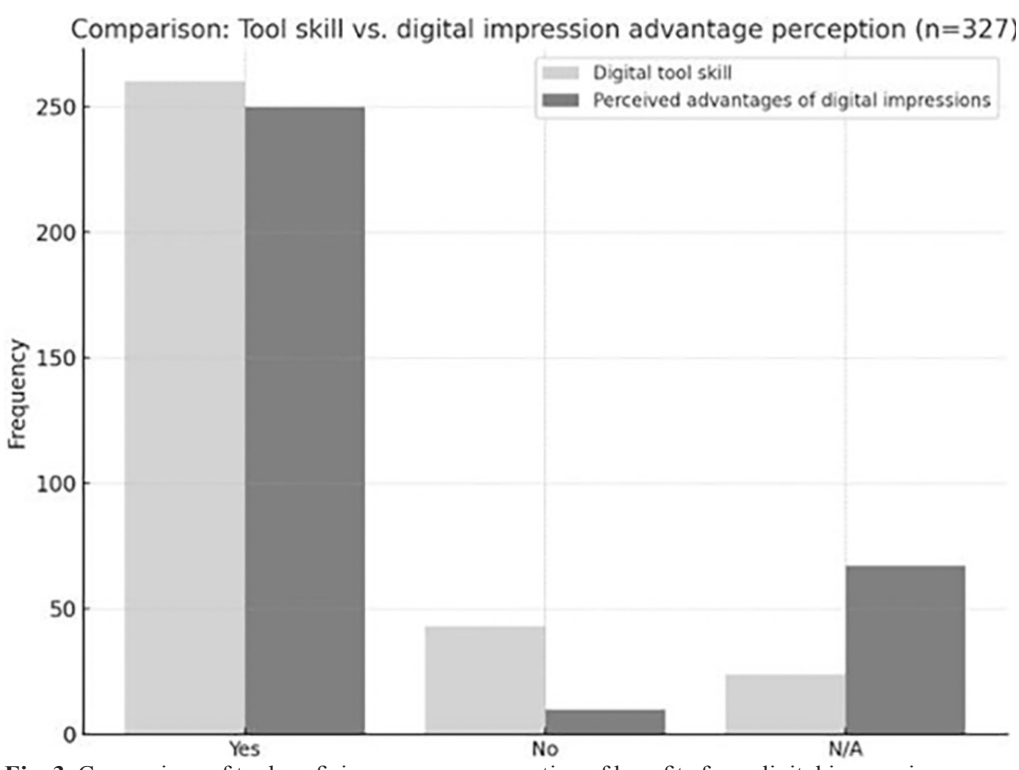
Comparison of tool proficiency versus perception of benefits from digital impressions.

Regarding the association between computer tool usage and the perceived advantages of digital workflows in dentistry, 62.4% (n = 204) of participants indicated that they believed such use provides clinical benefits, representing the largest proportion (Fig. 3).

3

**Figure 4:**
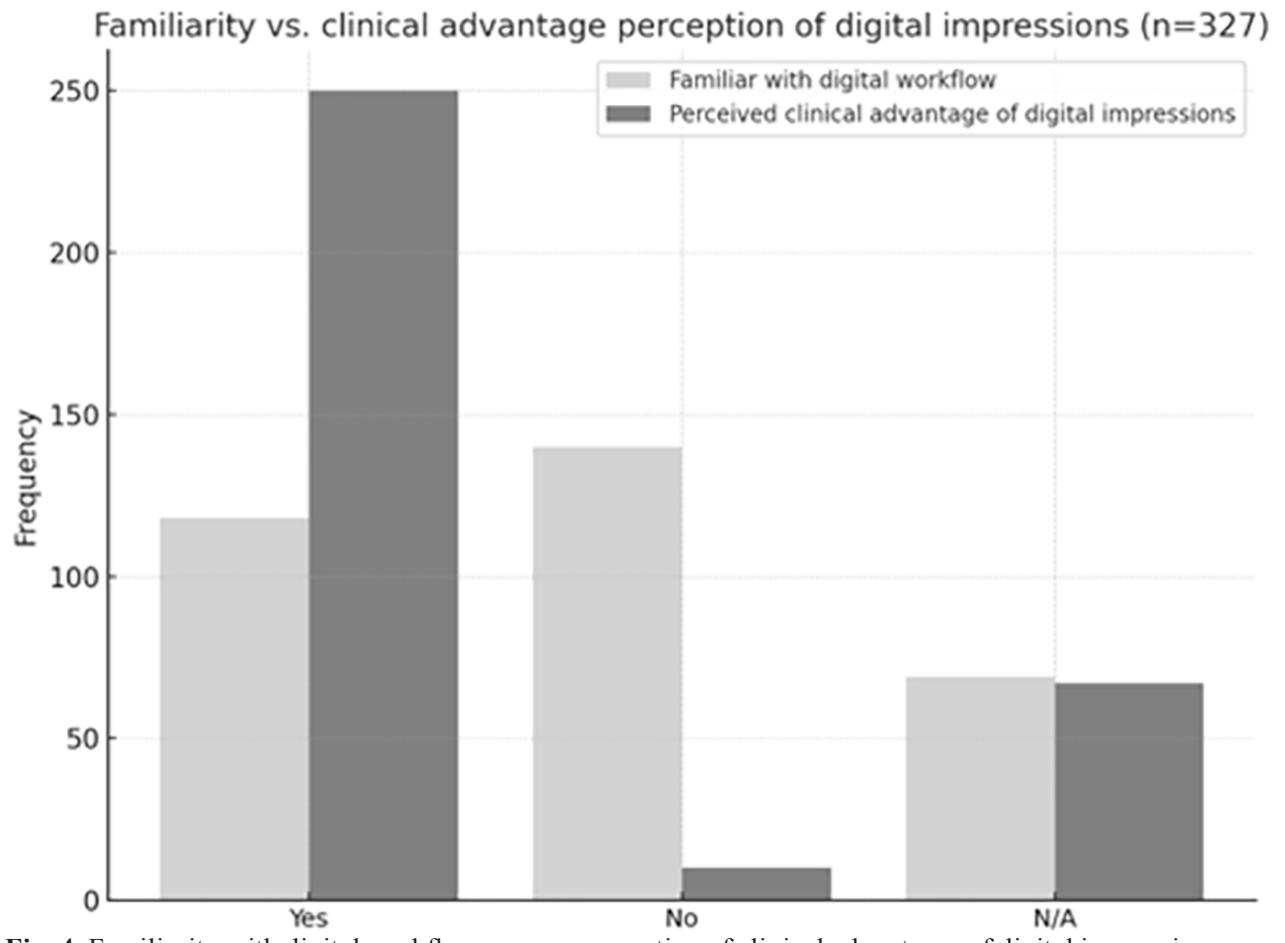
Familiarity with digital workflows versus perception of clinical advantages of digital impressions.

When examining respondents' familiarity with digital workflows and its relation to perceived clinical advantages, 30.9% (n = 101) reported being unfamiliar with digital workflows, while 14.1% (n = 46) selected "Don't know / No answer" (NS/NC) (Fig. 4).

4

In the analysis of the relationship between digital workflow training and learning with computers in the classroom, 34.3% (n = 112) of participants reported not having received any training in digital workflows, while 6.1% (n = 20) chose "Don't know / No answer" (NS/NC).

## Discussion

The results of this study underscore the need to incorporate knowledge of digital technologies into current dental hygiene training programs, a change that is likely to be positively received by students aged 16-19. The findings also demonstrate that students show greater receptivity to learning when technological elements are integrated into the educational process, fostering a more realistic and practical understanding of the subject matter (12). The use of a cross-sectional survey design was appropriate for exploring dental hygiene students' perceptions and knowledge regarding digital technologies. Including participants from various instructional modalities and employing a validated questionnaire enhanced both the representativeness and reliability of the study. Furthermore, the use of Chi-square tests and Cramer's V for statistical analysis allowed for identification of significant associations between key variables, reinforcing the methodological rigor. This approach is commonly applied in educational research for its effectiveness in capturing complex realities across diverse training contexts (13). A detailed analysis of the findings reveals a notable disparity in students' familiarity with essential digital dentistry concepts, such as digital flow and CAD/CAM systems. A significant proportion of students reported unfamiliarity with these terms, highlighting gaps in current academic training (14). This knowledge deficit may have implications for the professional preparedness of future graduates, potentially limiting their capacity to meet the demands of modern dental practice. Nonetheless, the expressed student interest in integrating digital flow into their curricula reflects a positive attitude toward curriculum modernization (15 - 16). Additionally, the broad recognition of the value of 3D technology for training further emphasizes the need to incorporate such tools to enhance both education and clinical practice (14). The perceived clinical advantages associated with digital workflows reinforce the urgency of integrating technological training to better equip future dental professionals (15 , 17). One striking finding is that 42.8% of participants were unfamiliar with the term "digital flow," while 21.1% responded with "Don't know / No answer" (NS/NC). This highlights a significant training gap that could adversely affect graduates' competencies (18). Moreover, 64.2% reported receiving no formal training in digital workflows, suggesting that despite the growing relevance of these technologies in practice, academic programs have yet to effectively incorporate them into their curricula (19). Similarly, 53.8% of respondents were unfamiliar with the term CAD-CAM, further underscoring the need to modernize educational content to better prepare students for the evolving technological landscape of dental practice (20). Despite these gaps, 89.3% of students expressed a clear interest in receiving training in digital workflows, indicating strong motivation to improve and update their knowledge. This willingness suggests that integrating digital technologies into academic programs would likely be well received and beneficial for student preparedness (18 - 19). Regarding perceptions of technology use, 44.3% of students believed that computer tools facilitate their learning, supporting the idea that integrating technology in education enhances both academic development and practical skill acquisition (14 , 20). Additionally, a substantial majority (81.7%) regarded 3D technology as beneficial to their academic training, highlighting the importance of including 3D modelling and simulation tools to improve diagnostic accuracy, treatment planning, and patient communication (21). The consistency of responses across different age groups and genders suggests that perceptions and knowledge of digital workflows and associated technologies are broadly similar within the study population, implying that curriculum improvements should be applied universally (22). These results align with international research emphasizing the benefits of early exposure to digital technologies in dental hygiene education. For example, the UTHealth study similarly reported high levels of student satisfaction and perceived usefulness of CAD/CAM tools despite limited prior experience (23). Likewise, an Indian study highlighted the importance of formal training to enhance student perceptions, consistent with our findings. Unlike these prior studies, our sample included diverse educational modalities (on-site, blended, and online) and focused on a socioeconomically disadvantaged population, underscoring the need for equitable access to digital resources. Overall, integrating CAD/CAM into dental curricula is essential to prepare students for contemporary clinical practice (9). This study has several strengths. The use of a cross-sectional questionnaire design enabled the collection of reliable and robust data from a relatively large sample of 327 participants, enhancing representativeness. The online administration facilitated access and ensured respondent privacy, which may improve data quality. Moreover, including questions specifically targeting understanding of core concepts like CAD/CAM and digital flow allowed for a detailed assessment of student familiarity, highlighting specific areas for curricular improvement. The application of rigorous statistical analyses such as Chi-square and Cramer's V further strengthens the study's scientific validity by evaluating associations and effect sizes (24 , 25). Finally, addressing both students' perceptions and training levels provides a comprehensive overview of the current educational landscape, offering valuable insights for educators and curriculum developers (22). However, the study also has limitations. It was conducted at a single vocational school in Seville, which may limit the generalizability of findings to other regions or academic contexts (26). The cross-sectional design precludes analysis of changes in perceptions or knowledge over time. Additionally, the sample was predominantly female, which could introduce gender bias. The absence of open-ended questions limited qualitative insight into students' nuanced perceptions and attitudes (21).

## Conclusions

Based on the analysis conducted, it is evident that there is an urgent need to incorporate training in digital technologies within the dental academic curriculum. The widespread lack of familiarity with fundamental concepts such as "digital workflow" and "CAD-CAM" among a significant proportion of students highlights a considerable gap in current educational preparation. Nonetheless, the overwhelmingly positive attitude of students-reflected by 89.3% expressing a desire to have digital workflow included in their training-is an encouraging indication for curriculum developers. This receptiveness suggests that updating the curriculum to integrate advanced technological tools and practices, including 3D modelling, would not only align with student expectations but also enhance their professional competence and readiness to navigate the evolving landscape of modern dentistry. Furthermore, the consistent perceptions across different age and gender groups emphasize the necessity of implementing these educational improvements inclusively and broadly across the student population.

## Figures and Tables

**Table 1:** “Familiarity with the concept of digital workflow”.

Response	Frequency	Percent	Valid Percent	Cumulative Percent
Yes	118	36.1%	36.1%	36.1%
No	140	42.8%	42.8%	78.9%
N/A	69	21.1%	21.1%	100.0%
Total	327	100.0%	100.0%	100.0%

1

**Table 2:** “Have you received training in digital workflow?”.

Response	Frequency	Percent	Valid Percent	Cumulative Percent
Yes	65	19.9%	19.9%	19.9%
No	210	64.2%	64.2%	84.1%
N/A	52	15.9%	15.9%	100.0%
Total	327	100.0%	100.0%	100.0%

2

**Table 3:** “Would you like to receive training in digital impression techniques (‘fingerprinting’) in future classes?”.

Response	Men	Women	Total
Yes	23 (7.0%)	269 (82.3%)	292 (89.3%)
No	2 (0.6%)	13 (4.0%)	15 (4.6%)
N/A	3 (0.9%)	17 (5.2%)	20 (6.1%)
Total	28 (8.6%)	299 (91.4%)	327 (100.0%)

3

**Table 4:** “Do you believe that the use of technologies such as digital tablets”.

Response	Men	Women	Total
Yes	14 (4.3%)	182 (55.7%)	196 (59.9%)
No	0 (0.0%)	21 (6.4%)	21 (6.4%)
N/A	14 (4.3%)	96 (29.4%)	110 (33.6%)
Total	28 (8.6%)	299 (91.4%)	327 (100.0%)

4

## Data Availability

The datasets generated and/or analyzed during the current study are available from the corresponding author upon reasonable request.
